# Using Logistic Regression to Predict Onset and Recovery With Tau Equivalency

**DOI:** 10.3389/fpsyg.2018.01849

**Published:** 2018-10-01

**Authors:** Kimmo Sorjonen, Michael Lundberg, Bo Melin

**Affiliations:** ^1^Department of Clinical Neuroscience, Karolinska Institutet, Stockholm, Sweden; ^2^Department of Public Health Sciences, Karolinska Institutet, Stockholm, Sweden

**Keywords:** Lord’s paradox, onset, recovery, simulation, tau equivalency

## Abstract

Many studies analyze the effect of a predictor X on the onset or recovery of an outcome Y, for example some kind of disorder. The findings from this simulation study indicate that such effects can be found even if there are no changes in individuals’ true scores on the outcome, i.e., with tau equivalency, given some degree of positive test-retest correlation of the outcome and a correlation between the predictor and the outcome at baseline. Researchers predicting onset/recovery should be aware of this fact and in order not to draw hasty conclusions to control for what can be expected from these correlations alone.

## Introduction

In some studies a dichotomous outcome, e.g., some kind of disorder, measured at a certain point in time is regressed, using logistic regression or survival analysis, on a predictor from an earlier time point (measured prospectively or retrospectively) while excluding individuals who experienced the disorder at an earlier time point from the analysis. The outcome may be dichotomous from the beginning or made dichotomous by using a cut-off on a continuous scale. If an effect is found in studies using this method, the predictor is assumed to have an association with onset of the outcome.

Focusing on depression, studies using this method have concluded, for example, that increased likelihood for onset of depression can be predicted from female sex (Barry et al., 2008; Eaton et al., 2008; Rudaz et al., 2017), increased levels of thyroid peroxidase antibodies during early gestation (Wesseloo et al., 2018), entering the perimenopause (Cohen et al., 2006), short alleles of the serotonin transporter gene (Eaton et al., 2008), diabetes mellitus (Zhang et al., 2017), higher neuroticism scores (Rudaz et al., 2017), irritability and fear/anxiety (Rice et al., 2017), loss and humiliation (Kendler et al., 2003), as well as low socioeconomic position (Kosidou et al., 2011).

In the other direction, studies that include only those above a cut-off at baseline, i.e., patients, have concluded, for example, that increased likelihood for recovery from depression can be predicted from female sex, being married, and less severe initial depression (Meyers et al., 2002), male sex (Barry et al., 2008), better family functioning, lack of comorbid illness, and shorter initial hospitalization (Keitner et al., 1992), lower degree of psychosocial impairment (Solomon et al., 2008), as well as treatment responsiveness (Curry et al., 2011).

We suspect that results similar to the ones mentioned above can be attained also in situations with tau equivalency. With tau equivalency there are no changes in individuals’ true scores on the outcome variable and all observed changes are due to random fluctuations around this true score. This suspicion is based on the following predictions: (1) Even without any change in their true score, some individuals will, due to random fluctuation, move above/below the cut-off, i.e., experience onset/recovery, from T0 to T1. Onset should be especially likely if the cut-off is set at a low level while recovery should be especially likely with a highly set cut-off; (2) With some degree of positive correlation between the outcome at T0 and T1, the likelihood of onset/recovery should be highest for those who are close to the cut-off at T0; If a predictor X has an association with the outcome at T0, those close to the cut-off at T0 will tend to have a higher/lower value on X than those further away from the cut-off. Consequently; (3) With some degree of positive correlation between the outcome at T0 and T1, if a predictor X has an association with the outcome at T0, there will be blood, or at least an association between X and the likelihood of onset/recovery. The objective of the present simulation study was to evaluate these predictions.

## Methods

Using R 3.3.2 statistical software (R Core Team, 2016), data was simulated through the following steps (script and data available as Supplementary Material or from https://osf.io/qb6mf/): (1) A number of virtual subjects were assigned a true score (T) on the outcome from a random normal distribution; (2) An observed value on the outcome at T0 and T1 (Y0 and Y1) were calculated for each subject by creating variables with a defined population correlation with T (same correlation for Y0 and Y1); (3) An observed value on a predictor X was calculated for each subject by creating a variable with a defined population correlation with Y0; (4) Y0 and Y1 were dichotomized by using a cut-off. The following parameters were calculated and saved in a data frame: (a) The correlation between Y0 and Y1 as well as between Y0 and X, both below and above the cut-off; (b) The proportion experiencing onset/recovery between T0 and T1; (c) The effect of Y0 on the natural logarithm of the odds (=logit) to experience onset/recovery between T0 and T1, calculated with logistic regression; (d) The effect of X on the logit for onset/recovery. These steps were repeated 200 times for each combination of 6 different sample sizes (between 500 and 121.500), 7 different population correlations between T and Y0/Y1 (between 0 and 0.9), 7 different population correlations between Y0 and X (between −0.9 and 0.9), and 7 different cut-offs (between 1.8 *SD* below and 1.8 *SD* above the mean), resulting in 411.600 data sets. Data sets where the proportion of onset or recovery was 100% (*n* = 4174) were deleted from the analyses. In some cases the effects of Y0 and X on the logit for onset/recovery were very extreme, so these variables were trimmed by deleting 2.5% of the lowest and 2.5% of the highest values. The three predictions (see above) were evaluated with linear regression analyses, in the case of predictions 2 and 3 with coefficients from logistic regression as the outcome. It should be emphasized that this data simulates a situation where all subjects’ have exactly the same true score on the outcome at T0 and T1 and that all intra-individual differences between the observed values Y0 and Y1 are due to random fluctuations around this true score.

## Results

The results are presented in the order of the predictions above: (1) The logit for onset/recovery, without any change in true scores, was influenced by used cut-off (standard deviations from the mean). However, this effect was moderated by the test-retest correlation (*r*_y0y1_bco_ and *r*_y0y1_aco_ for those below/above the cut-off at T0, respectively) according to the following (*R*^2^ = 0.96 for both predictions):

$$\begin{array}{lll} \mathrm{logit}(\mathrm{onset}) & = & \;-0.116-2.196\times r_{y0y1\_\mathrm{bco}}-1.596\\ & & \times \mathrm{cut-off}+1.974\times r_{y0y1\_\mathrm{bco}}\times \mathrm{cut-off}\\ \mathrm{logit}(\mathrm{recovery}) & = & \;-0.120-2.186\times r_{y0y1\_\mathrm{aco}}+1.590\\ & & \times \mathrm{cut-off}-1.953\times r_{y0y1\_\mathrm{aco}}\times \mathrm{cut-off} \end{array}$$

In accordance with predictions, the level of the cut-off has a negative effect on the probability of onset and a positive effect on the probability of recovery, but these effects diminish as the test-retest correlation for the outcome increases. For example, with a cut-off = 1.5 (*SD* above the mean) and a test-retest correlation = 0.7 (only including those below the cut-off at T0) the predicted logit(onset) = −0.116 – 2.196 × 0.7 – 1.596 × 1.5 + 1.974 × 0.7 × 1.5 = −1.975 and predicted probability for onset = e^−1.975^ / (1 + e^−1.975^) = 0.122.

(2) The coefficient for the effect of the outcome at T0 (Y0) on the logit for onset/recovery was influenced by the test-retest correlation according to the following (*R^2^* = 0.89 for both predictions):

$$\begin{array}{lll} \beta_{y0-\mathrm{logit}(\mathrm{onset})} & = & -0.0235+2.065\times r_{y0y1\_\mathrm{bco}}\\ \beta_{y0-\mathrm{logit}(\mathrm{recovery})} & = & 0.0234-2.063\times r_{y0y1\_\mathrm{aco}} \end{array}$$

In accordance with predictions, the closer people are to the cut-off at T0, the higher is the probability that they will experience onset/recovery, at least if there is some degree of positive test-retest correlation for the outcome. For example, with a test-retest correlation = 0.7 (only including those below the cut-off at T0) the predicted β_y0-logit(onset)_ = −0.0235 + 2.065 × 0.7 = 1.422. This means that for every increase in Y0 with one *SD* (i.e., the closer people are to the cut-off at T0) there is a fourfold increase (e^1.422^ = 4.145) in the odds to experience onset between T0 and T1.

(3) The coefficient for the effect of predictor X on the logit for onset/recovery was influenced by test-retest correlation, the correlation between X and the outcome at T0, as well as by the interaction of these two correlations, according to the following (*R^2^* = 0.78 for both predictions):

$$\begin{array}{lll} \beta_{x-\mathrm{logit}(\mathrm{onset})} & = & -0.00230+0.00179\times r_{y0y1\_\mathrm{bco}}+0.0110\\ & & \times r_{y0x\_\mathrm{bco}}+1.670\times r_{y0y1\_\mathrm{bco}}\times r_{y0x\_\mathrm{bco}}\\ \beta_{x-\mathrm{logit}(\mathrm{recovery})} & = & 0.00188-0.000966\times r_{y0y1\_\mathrm{aco}}-0.00772\\ & & \times r_{y0x\_\mathrm{aco}}-1.675\times r_{y0y1\_\mathrm{aco}}\times r_{y0x\_\mathrm{aco}} \end{array}$$

In accordance with predictions, a predictor X tends to have an association with the probability of onset/recovery when there is some degree of correlation between X and the outcome at T0 and some degree of positive test-retest correlation for the outcome. We also see that the effect of one of these correlations strengthen when the other correlation increases. For example, with a test-retest correlation = 0.7 (only including those below the cut-off at T0) and a correlation between X and Y0 = 0.5 (only including those below the cut-off at T0) the predicted β_x−logit(onset)_ = −0.00230 + 0.00179 × 0.7 + 0.0110 × 0.5 + 1.670 × 0.7 × 0.5 = 0.589. This means that for every increase in X with one *SD* there is an increase with 80% (e^0.589^ = 1.802) in the odds to experience onset between T0 and T1.

The standard error of the coefficient for the effect of X on the logit for onset/recovery was calculated for various combinations of sample size, test-retest correlation, and degree of correlation between X and the outcome at T0. The natural logarithm (in order to never predict a negative standard error) of these were predictable according to the following (*R^2^* = 0.47 for both predictions):

$$\begin{array}{lll} \log(\mathrm{SE}(\beta_{x-\mathrm{logit}(\mathrm{onset})})) & = & -1.748-0.00396\times \mathrm{sqrt}(N)\\ & & -0.372\times r_{y0y1\_\mathrm{bco}}\\ \log(\mathrm{SE}(\beta_{x-\mathrm{logit}(\mathrm{recovery})})) & = & -1.746-0.00394\times \mathrm{sqrt}(N)\\ & & -0.396\times r_{y0y1\_\mathrm{aco}} \end{array}$$

**FIGURE 1:**
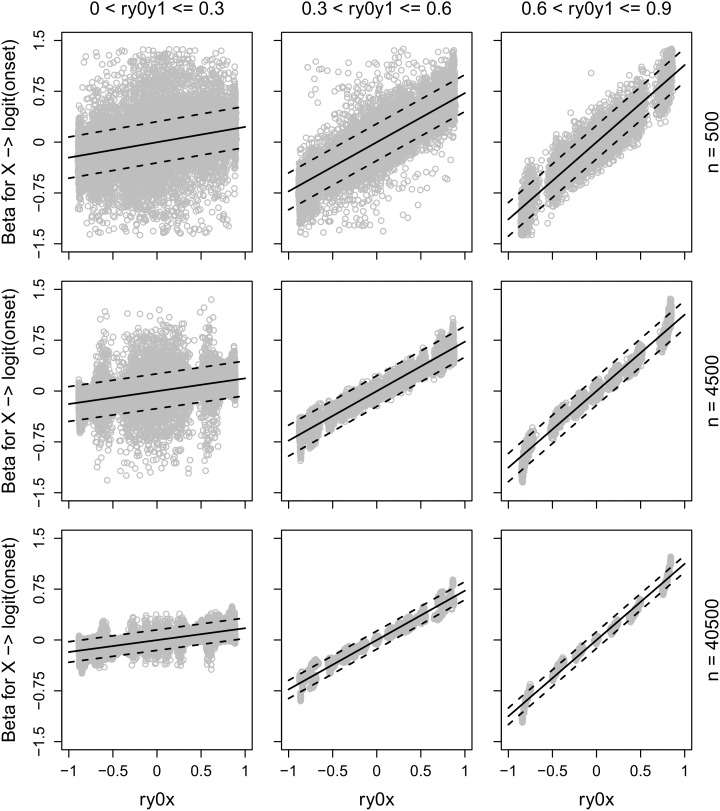
The association between the correlation between a predictor X and the outcome at T0 (ry0x) and the coefficient for the effect of X on the logit of onset on the outcome (=being above the cut-off) at T1, when there are no changes in individuals’ true scores (=tau equivalency), separately for three different ranges of test-retest correlation for the outcome (ry0y1) and three different sample sizes. The solid lines show predicted effects of X and the dotted lines show 95% CI.

The association between the correlation between a predictor X and the outcome at T0 and the coefficient for the effect of X on the logit for onset is presented in **Figure 1**, for the combinations of three different ranges of test-retest correlations for the outcome and three different sample sizes. The predicted effect of X, as given by the formulas above, is included as solid lines in the plots. A 95% CI was calculated by adding/subtracting two predicted standard errors to/from the predicted effect and these are included as dotted lines in the plots. Plots with the effect of X on the logit of recovery would look very similar, although the associations would be in the opposite direction.

## Discussion

In accordance with predictions, the effect of a predictor X on the logit for onset/recovery had an association with the test-retest correlation for the outcome and the correlation between X and the outcome at T0. Researchers predicting onset/recovery should be aware of these influences, and we recommend to control if the calculated effect of X on the logit of onset/recovery is outside the CI of what can be expected from the test-retest correlation and the correlation between X and the outcome at T0 alone (a function is available in the Supplementary Script). If this is not the case, researchers should think twice before drawing conclusions about an effect of X on onset/recovery, at least if they with onset/recovery mean something over and above random fluctuation.

The present finding might be seen as related to Lord’s paradox, a name given to the phenomenon that the effect of a predictor X on the change in an outcome (Y1 – Y0) can look very different if the baseline value of the outcome (Y0) is controlled for or not (Lord, 1967, see also van Breukelen, 2013; Pearl, 2016). To borrow an example from Sorjonen et al. (2017), imagine that a group of professional dart players get an average score of 9 and a group of amateurs an average score of 4 on two consecutive throws. Hence, the average change is zero in both groups and there is no effect of group on this change. However, would we include the score from the first throw as a covariate in the analysis, we would probably observe a positive effect of “professionalism” on the change in the score. The reason for this is that in the latter analysis the calculated effect is conditional on professionals and amateurs having the same score on the first throw, but if they do we can assume a more positive measurement error (luck) for amateurs and a more negative measurement error (bad luck) for professionals. As luck/bad luck tends to even out in the long, or even the short, run, we can expect professionals who got the same score as amateurs on the first throw to have a more positive change from the first to the second throw.

So, the combination of an association between X and Y at baseline, which tends to result in an association between X and measurement error given the same value on Y at baseline, and less than perfect reliability in the measurement of Y, which tends to result in regression toward the mean, gives us Lord’s paradox (Eriksson and Häggström, 2014). The present finding, that X can have an effect on the probability of onset/recovery of Y even without any change in individuals’ true scores, is due to the fact that if X has an association with Y at baseline then those close to the cut-off tend to have a higher/lower value on X than those further away from the cut-off, and closeness to the cut-off at baseline is predictive of experiencing onset/recovery between the measurements, at least with some degree of positive test-retest correlation for the outcome.

The present study is limited by the fact that all complexities and nuances of real life are impossible to incorporate into a simulation study. For example, we used variables drawn from normal distributions although many variables used in clinical research tend to be skewed. If and how the association between a predictor X and the onset/recovery of an outcome Y is moderated by the skewness of the predictor and the outcome could be a suitable objective for a future study. It could also be interesting to study if other types of analyses, for example employing machine learning algorithms, are affected by the same kind of bias as logistic regression as demonstrated in the present paper.

## Author Contributions

KS carried out the simulations and analyses and wrote an initial draft. KS, ML, and BM conceived of the presented idea discussed the results and contributed to the final manuscript. All authors have approved the final version of the manuscript.

## Conflict of Interest Statement

The authors declare that the research was conducted in the absence of any commercial or financial relationships that could be construed as a potential conflict of interest.
